# Hepatobiliary disease in XLMTM: a common comorbidity with potential impact on treatment strategies

**DOI:** 10.1186/s13023-021-02055-1

**Published:** 2021-10-12

**Authors:** Adele D’Amico, Antonella Longo, Fabiana Fattori, Michele Tosi, Luca Bosco, Maria Beatrice Chiarini Testa, Maria Giovanna Paglietti, Claudio Cherchi, Adelina Carlesi, Irene Mizzoni, Enrico Bertini

**Affiliations:** 1 Unit of Muscular and Neurodegenerative Disorders, Genetics and Rare Diseases Research Division, Department of Neurosciences, Bambino Gesù Children’s Hospital, IRCCS, piazza S. Onofrio 4, 00165 Rome, Italy; 2 Genetics and Rare Diseases Research Division, Bambino Gesù Children’s Hospital, IRCCS, Rome, Italy; 3 Pneumology Unit, University Hospital Pediatric Department, Bambino Gesù Children’s Hospital, IRCCS, Rome, Italy; 4 Unit of Neurorehabilitation, Department of Neuroscience, IRCCS Bambino Gesù Children’s Hospital, Rome, Italy; 5 Unit of Child Neurology and Psychiatry, Tor Vergata University Hospital, Rome, Italy

**Keywords:** Myotubular myopathy, XLMTM, Hepatobiliary

## Abstract

**Background:**

X-linked myotubular myopathy (XLMTM) is a rare congenital myopathy resulting from pathogenic variants in the *MTM1* gene. Affected male subjects typically present with severe hypotonia and respiratory distress at birth and they often require intensive supportive care. Long-term survivors are often non-ambulant, ventilator and feeding tube–dependent and they generally show additional organ manifestations, indicating that myotubularin does play a vital role in tissues other than muscle. For XLMTM several therapeutic strategies are under investigation. For XLMTM several therapeutic strategies are under investigation including a study of intravenous MTM1 gene transfer using a recombinant AAV8 vector of which has some concerns arises due to hepatotoxicity.

**Results:**

We report prospective and retrospective clinical data of 12 XLMTM patients collected over a period of up to 10 years. In particular, we carried out a thorough review of the data about incidence and the course of hepatobiliary disease in our case series.

**Conclusions:**

We demonstrate that hepatobiliary disease represents a common comorbidity of XLMTM that seems irrespective to age and diseases severity. We recommend to carefully explore and monitor the hepatobiliary function in XLMTM patients. We believe that a better understanding of the pathogenic mechanisms that induce hepatobiliary damage is essential to understand the fatal events that may occur in the gene therapy program.

## Background

X-linked myotubular myopathy (XLMTM, OMIM #310400) is a rare congenital myopathy resulting from pathogenic variants in the *MTM1* gene. *MTM1* encodes the protein myotubularin, a lipid phosphatase involved in the maintenance of skeletal muscle structure and membrane homeostasis, in particular in the organization of transverse tubules [1–5]. Moreover, a block in autophagic degradation has been also demonstrated in mouse *MTM1* models [6].

XLMTM has been classified in mild, intermediate, or severe forms based on the amount of the ventilator support required [7].

The severe form represents the most common phenotype. Affected babies typically present with marked hypotonia and respiratory distress at birth, and they require respiratory and feeding intensive support. Long-term survivors are mostly non-ambulant and ventilator and feeding tube-dependent. XLMTM is also characteristically complicated by systemic diseases including genitourinary disorders, liver dysfunction, spherocytosis and bleeding diathesis [8–12].

The development of different animal models and their characterization has greatly contributed to the understanding of the clinical and pathophysiological aspects of XLMTM and has been essential in the advance of therapeutic approaches [13].

For XLMTM several therapeutic strategies are nowadays under investigation, such as adeno-associated virus (AAV)-mediated gene replacement therapy (NCT03199469), enzyme replacement, dynamin-2 modulation and PIK3C2B inhibition [3, 14–18].

Preclinical studies of gene replacement were conducted in animal models of XLMTM, demonstrating that the systemic administration of recombinant AAV-8 vector expressing the MTM1 transgene is well-tolerated in canine model without adverse events even at high dose (up to 8 × 10^14^ vg/kg) as well as in wild-type non-human primates [19, 20]. In light on these encouraging results, the gene replacement approach has been translated in humans. ASPIRO is a first in-human study of intravenous MTM1 gene transfer using a recombinant AAV8 vector (AT132).

To date, 17 boys have received AT132 in the initial dose escalation cohort or in a later expansion cohort. Unexpectedly, three patients of the later expansion cohort who received the highest dose experienced a fatal liver dysfunction. In these three patients the liver findings, after treatment with AT132, included intrahepatocellular and canalicular cholestasis, bile ductular reaction, secondary fibrosis, and notable lack of prominent liver parenchymal inflammatory cellular infiltrates [20]. All these boys had evidence of likely pre-existing intrahepatic cholestasis and, although full investigations to elucidate the cause of these fatal events are still ongoing, cholestasis may have played a pathogenic role.

Hepatobiliary disease, including gallstones and peliosis hepatis, is a well-known comorbidity of XLMTM, and has been reported in several case series since the 90’s. Retrospective and prospective cohort’s studies documented different percentages of this complication [8–11]. Amburgey and colleagues have reported abnormal liver enzymes, enlarged liver and jaundice respectively in 22.5%, 11.8% and 14.7% in their cohort, as well as liver bleed/haemorrhage and gallstones in 5.9% and 8.8% of patients, respectively [8]. In the retrospective Recensus clinical study, hepatobiliary disorders were reported in 7% of patients [9]. Similar percentages were reported in NatHis prospective study [11] and in the retrospective study published by Herman and colleagues in which the hepatobiliary complication were reported in less than 10% of patients [10].

As part of our efforts to contribute to a deep knowledge of this comorbidity, we reviewed in detail the long-term follow-up of 12 XLMTM patients. In our cohort, the hepatobiliary function was systematically assessed through blood chemistry and liver ultrasound, regardless of clinical symptoms. We expected that this clinical approach could provide more accurate data on hepatobiliary disease in XLMTM and may give more insight on management of gene therapy.

## Methods

The study group consisted of 12 XLMTM patients followed up at the Unit of Muscular and Neurodegenerative Diseases of the Bambino Gesù Children’s Hospital between the years 2012 and 2021. All patients have a genetic defect in *MTM1*, identified by direct genomic sequencing.

We retrospectively reviewed clinical records for demographic data, clinical features and other disease-related comorbidities. Some of these patients (pts #1 to #7 in Table 1) were also enrolled in the international prospective and longitudinal natural history study “NatHis-MTM” between March 2015 and September 2020 (NCT02057705).

**Table 1:** Clinical, laboratory and instrumental data of patients’s cohort

Patient	#1	#2	#3	#4	#5	#6	#7	#8	#9	#10	#11	#12
Age at diagnosis	1 m	27 d	1 m	17 d	2 m	3 y	8 m	29 d	1 m	28 d	1 m	28 d
Age at last visit	5 y	8 y	19 y	9 y	9 y	10 y	1 y	2 y	2 y	7 m	7 m	10 m
Age at tracheostomy	56 d	60 d	224 d	NIV	77 d	187 d	NIV	No	NIV	29 d	125 d	No
Hour of ventilation	24	24	10	10	12	14	9	12	8	24	10	No
Age at gastrostomy	1 m	2 m	7 m	1y, 10 m	5 m	6 m	8 m	2 m	7 m	29 d	24 d	3 m
Cholelithiasis, (age at first observation)	Yes (25 m)	No	No	Yes (22 m)	Yes (47 m)	Yes (100 m)	No	No	yes (18 m)	No	No	No
Transaminases	Elevated	Elevated	nv	nv	nv	elevated	nv	nv	elevated	nv	nv	elevated
Liver echo	Dishom.	Hyper echogenic	No	Dishom.	Dishom.	Peliosis, dishom.	No	No	dishom.	Peliosis, dishom.	No	No
Nephrolithiasis	yes	Yes	No	Yes	Yes	No	No	No	No	No	Yes	No
Cryptorchidism	Yes	Yes	No	Yes	Yes	Yes	Yes	Yes	No	yes	Yes	yes
Ophtalmoplegia	Yes	Yes	Yes	Yes	Yes	Yes	No	Yes	yes	No	No	No
Head control	No	20 m	24 m	24 m	12 m	10 m	No	14 m	21 m	No	No	6 m
Sitting position	No	24 m (lost 36 m)	27 m	No	50 m	14 m (lost 9 y)	No	18 m	26 m	No	No	8 m
Walking	No	No	30 m (lost 13 y)	No	No	No	No	No	No	No	No	No
Scoliosis	No	30°	> 60°	47°	40°	>60°	No	No	No	No	No	No
Coagulation or hematologic defect	No	No	No	No	thrombosis	No	No	No	No	No	No	No

The table summarize the clinical, biochemical and instrumental findings of our 12 XLMTM patients

*y* years, *m* months, *d* days, *NIV* non invasive ventilation, *nv* no value available, *dishom*. dishomogeneity

Each patients since diagnosis was evaluated every six months or more frequently according to clinical needs. At each visit we performed a complete physical examination, including motor, respiratory and bulbar assessments as well as blood chemistries for liver, kidney, coagulation, and hematologic functions. A standard liver ultrasound was performed every year.

The clinical phenotypes of the patients were defined according to the clinical classification published by McEntagart in 2002 [7] as a mild phenotype (no ventilatory support), an intermediate phenotype (ventilatory support less than 12 h a day) or a severe phenotype (ventilation support 12 or more hours a day).

The study was conducted in accordance with the Declaration of Helsinki. All patients and/or parents of minors signed consent forms for participation to this research observational study and for data publication. This study was approved by local EC.

## Results

### Patients

Twelve male patients aged from 0.17 months to 18.61 years were included in the study. Clinical finding are summarized in Table 1. One of these patients died at 23 months of age due to sepsis (pt#7). All patients presented with genetically confirmed pathogenic *MTM1* variants.

### Genetic data

Pathogenic variants identified in our cohort of patients, consisting of truncating, missense, large deletions and splicing mutations, were spread throughout the different domains of the myotubularin. Two patients had a deletion of a single nucleotide (pt #8) or several bases (pt #1) resulting in a frameshift mutation. Four cases showed an in frame deletion of one or two amino acids in PH-GRAM (pt #2–4 and #6) or RID (pt #5) domain of myotubularin. In patient #2 the in-frame deletion of six base pairs in exon 3 (p.33_34delGluAla) was associated with a large deletion encompassing exon 4. A large deletion from exon 1 to exon 9 of MTM1 was also found in patient #9. Three different missense mutations (p.R69C, p.R241C and p.I185K) were identified in patients #3, #7 and #12 respectively. Patient #10 had a truncating variant in RID domain and patient #11 carried a splicing alteration leading to two different aberrant transcripts.

Precise pathogenic variants are listed in Table 2. The variants shown are described using the *MTM1* gene NM_000252.3 transcript reference sequence.

**Table 2:** Pathogenetic variants found in our cohort

Patient	Nucleotide change	Aminoacid change
1	c.99_105delGGCTGTT	p.Glu33Aspfs*9
2	c.98_103delAGGCTG + Exon 4 deleted	p.33_34delGluAla + p.?
3	c.205 C>T	p.Arg69Cys
4	c.139_141delAAA	p.Lys47del
5	c.564_566delTAA	p.Asn189del
6	c.139_141delAAA	p.Lys47del
7	c.721 C>T	p.Arg241Cys
8	c.1138delG	p.Asp380Thrfs*6
9	Exon 1–9 deleted	p.?
10	c.664 C>T	p.Arg222*
11	c.1261-5T>G	p.Arg421_Gln451del + p.Arg421Serfs*7
12	c.554T>A	p.Ile185Lys

In this table are summarized the genomic variants and aminoacid changes found in our patients

### Clinical characteristics

Our cohort consists of 12 patients: 8 patients had intermediate phenotype (66%) and 4 (34%) had a severe phenotype.

All patients manifested with generalized hypotonia and respiratory failure at birth that required ventilatory support. Seven patients underwent tracheostomy at a median age of 77 days of life (range 29–244), while five required a non-invasive ventilatory support. Most ventilated patients gained some hours of ventilator independence over time.

All patients needed feeding support from birth, and they all underwent gastrostomy at mean age of 5 months (range 1–22). Five of them (41%) achieved over time the ability to eat small semisolid meals.

All patients achieved, albeit belatedly, some motor milestones (details are reported in Table 1). In summary, head control was achieved in 8/12 patients, while 7 patients reached the ability to sit independently. One patient achieved the ability to walk independently, but it was subsequently lost in puberty. In the long-term follow up we documented a motor deterioration in some patients who lost some of the motor milestones previously achieved. Progressive scoliosis was observed in all patients by the age of 5 years, exceeding a Cobb angle of 40° at the mean age of 8 years.

### Related conditions

Other medical conditions considered related to XLMTM are reported in Table 1. In general, ophtalmoplegia, renal lithiasis and cryptorchidism were observed in most patients, whereas bleeding diathesis, congenital spherocytosis or endocrine dysfunction were absent in our patients.

### Hepatobiliary disorders

Hepatobilary disorders were observed in about half of our case series as reported in Table 1.

**Fig. 1:**
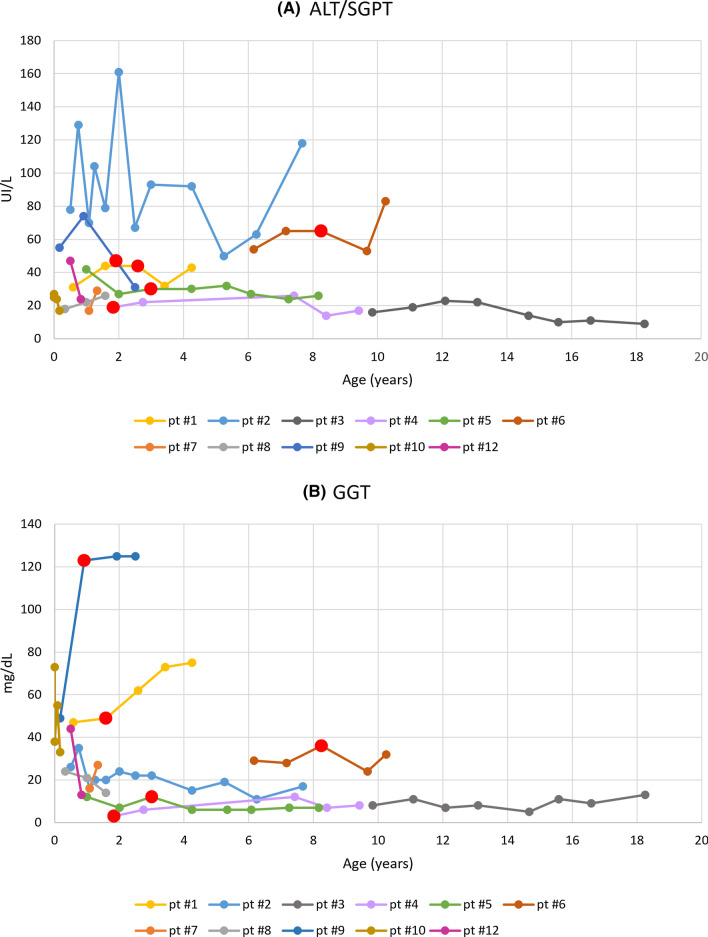
The figure shows the trend over time of values of alanine transaminase (ALT, panel **A**) and gamma-glutamyltransferase (GGT, panel **B**). Patients #1, #2, #6, #9 presented with transient or persistent elevations in serum aminotransferases. Patient #12 not shown in Fig. 1 because only two measurements were available. Red dots indicate, for each patient, the first occurrence of cholelithiasis

Abnormal liver structure (including higher or abnormal echogenicity or blood-filled cysts) was found in 7/12 patients (58%), whereas high levels of serum transaminases were documented in 5/12 patients (42%) (Fig. 1). In 5 (42%) patients we documented gallstones that were asymptomatic in 4 of them. Two patients had blood-filled cysts within the liver compatible with peliosis hepatis. One of them also manifested spontaneous liver bleeding at the age of 4 years. Indeed, only one patient (pt #9) manifested three intermittent episodes of itching cholestatic jaundice. The first long term episode was triggered by vaccination for hepatitis at the age of 18 months. Two days after Hep B vaccination he manifested jaundice and pruritus associated to severe increase of serum conjugated bilirubin (up to 15 mg/dl), liver enzymes (AST 118 UI/l), gamma-glutamyl transferase (GGT 60 mg/dl) and biliary acids (322 µM/L; range 0.00–6.00.) Liver MRI showed normal-sized liver with preserved structure and a small gallstone in the absence of abnormalities in the intrahepatic and extrahepatic bile ducts. Extensive laboratory tests excluded hepatotrophic viral infections. Pharmacologic treatment with ursodeoxycholic acid (UDCA) was initiated. Within a month, there was a resolution of jaundice and pruritus and progressive normalization of serum bilirubin associated to a slight reduction of biliary acids, transaminase and GGT. Since then, over the course of a year, the child presented two additional episodes of jaundice of lower intensity and short duration.

## Discussion

We report on clinical findings and long-term follow up of 12 XLMTM patients with the aim to describe the systemic complication of this disease.

Above all, our intention was to better define and describe the frequency of hepatobiliary comorbidity, quite often asymptomatic, in these fragile patients.

In our opinion, this aspect deserves particular attention in the light of recent and fatal events occurred in the three oldest and heaviest patients enrolled in the ASPIRO trial who have received the higher dosage of AT132 (3 × 10^14^ vg/kg).

Although the pathological mechanisms that lead to liver dysfunction are not yet fully understood, it is likely that hepatobiliary disease might have contributed to the fatal outcome.

In our case series high levels of serum transaminases with gallstones and abnormal liver structure were detected, respectively, in 42% and 58% of patients.

These results showed a higher incidence of hepatobiliary disturbances than has been reported in other retrospective and protective studies [8–11].

In our observational study the laboratory and instrumental examinations have been systematically carried out to investigate the liver function, regardless of symptoms.

The longitudinal analysis of our data seems to suggest that hepatobiliary disease is a common and not progressive condition and that it does not correlate with age or disease duration, nor with clinical severity or type and site of *MTM1* mutation. Indeed, our oldest patient (pt#3) do not manifest any liver abnormalities whereas the most serious episode of cholestasis occurred in one of the youngest child in our series. To date, it remains unknown why XLMTM patients have multisystem manifestations including the hepatobiliary disorders and the underlying pathophysiological mechanisms are still not clear.

Myotubularin is the archetypal member of the MTM family of lipid phosphatases which dephosphorylate phosphatidylinositol 3-monophosphate (PI3P) and phosphatidylinositol 3,5-bisphosphate (PI(3,5)P2), and it plays a role in lipid membrane trafficking from the late endosome to the lysosome, in vacuolar formation and morphology, and it also regulates desmin intermediate filament assembly and architecture [21–23].

Moreover, it has also been demonstrated that autophagy is compromised in skeletal muscle of mice MTM1-deficient mice through the hyperactivation of the mTORC1 signaling [6]. Because the deregulation of autophagy has been also linked to many liver diseases including cholestasis [24, 25], we suggest that it may also have a role in the hepatobiliary disorders of XLMTM patients.

In conclusion, the long-term clinical observation in our cohort confirm that hepatobiliary disease is a common complication of XLMTM that we observed in a higher percentage of patients than previously reported. We strongly recommend carefully exploring and monitoring the hepatobiliary function in XLMTM patients. The early recognition of this complication may help the patient’s management, both for ordinary care and for any experimental treatment.

Finally, we believe that a better understanding of the pathogenic mechanisms that induce hepatobiliary damage would be essential to understand the fatal events that may occur in the gene therapy program.

## Data Availability

The datasets used during the current study are available from the corresponding author on reasonable request. All data relevant to the study are included in the article.
