# Correction to: Hepatobiliary disease in XLMTM: a common comorbidity with potential impact on treatment strategies

**DOI:** 10.1186/s13023-021-02171-y

**Published:** 2022-01-17

**Authors:** Adele D’Amico, Antonella Longo, Fabiana Fattori, Michele Tosi, Luca Bosco, Maria Beatrice Chiarini Testa, Maria Giovanna Paglietti, Claudio Cherchi, Adelina Carlesi, Irene Mizzoni, Enrico Bertini

**Affiliations:** 1 Unit of Muscular and Neurodegenerative Disorders, Genetics and Rare Diseases Research Division, Department of Neurosciences, Bambino Gesù Children’s Hospital, IRCCS, piazza S. Onofrio 4, 00165 Rome, Italy; 2 Genetics and Rare Diseases Research Division, Bambino Gesù Children’s Hospital, IRCCS, Rome, Italy; 3 Pneumology Unit, University Hospital Pediatric Department, Bambino Gesù Children’s Hospital, IRCCS, Rome, Italy; 4 Unit of Neurorehabilitation, Department of Neuroscience, IRCCS Bambino Gesù Children’s Hospital, Rome, Italy; 5 Unit of Child Neurology and Psychiatry, Tor Vergata University Hospital, Rome, Italy

## Correction to: Orphanet J Rare Dis (2021) 16:425 10.1186/s13023-021-02055-1

Following publication of the original article [1], the authors reported that the given name of Maria was omitted from the author group. The given name has been added to the author group and is presented correctly in this correction article.

The incorrect author name is: Giovanna Paglietti.

The correct author name is: Maria Giovanna Paglietti.

The original article [1] has been corrected.
